# The Evolution of the Physician Role in the Setting of Increased Non-physician Clinicians in Sub-Saharan Africa: An Insistence on Timing and Culturally-Sensitive, Purposefully Selected Skill Development: Comment on "Non-physician Clinicians in Sub-Saharan Africa and the Evolving Role of Physicians"

**DOI:** 10.15171/ijhpm.2016.90

**Published:** 2016-07-09

**Authors:** Agnes Binagwaho, Gabriela Sarriera, Arielle Eagan

**Affiliations:** ^1^Minister of Health, Kigali, Rwanda.; ^2^Harvard Medical School, Boston, MA, USA.; ^3^Geisel School of Medicine, Dartmouth University, Hanover, NH, USA.; ^4^University of Global Health Equity, Kigali, Rwanda.; ^5^University of Vermont, Burlington, VT, USA.; ^6^The Dartmouth Institute of Health Policy and Clinical Practice, Hanover, NH, USA.

**Keywords:** Non-physician Clinicians (NPCs), Physicians, Task-Shifting, Sub-Saharan Africa, Non-clinical Skills, Rwanda

## Abstract

As Eyal et al put forth in their piece, *Non-physician Clinicians in Sub-Saharan Africa and the Evolving Role of Physicians,* task-shifting across sub-Saharan Africa through non-physician clinicians (NPCs) has led to an improvement in access to health services in the context of physician-shortages. Here, we offer a commentary to the piece by Eyal et al, concurring that physician’s roles should evolve into specialized medicine and that skills in mentorship, research, management, and leadership may create more holistic physicians clinical services. We believe that learning such non-clinical skills will allow physicians to improve the outcome of their clinical services. However, at the risk of a local, clinical brain drain as physicians shift to explore beyond the clinical sphere, we advocate strongly for increased caution to be exercised by leadership over the encouragement of this evolution. In the context of still-present physician shortages across many developing countries, we advocate to analyze this changing role and to purposefully select each new skill according to the context, giving careful consideration to the timing and degree of its evolution.

In 2006, sub-Saharan Africa held 24% of the disease burden in the world but only a startling 4% percent of the global health workforce.^1^ Today, despite advancements in health workforce capacity building, this imbalance remains.^2^ It is in this context of strained human resources that Eyal et al take their position in *Non-physician Clinicians in Sub-Saharan Africa and the Evolving Role of Physicians*, discussing new coverage of once-physician-driven clinical areas by non-physician clinicians (NPCs) and advocating for increased non-clinical education for doctors to prepare them to enter new roles beyond traditional medical fields. In response to Eyal et al, we stress the need to carefully analyze and weigh the influence of clinician redistribution across non-clinical fields before advocating for the evolution of physicians’ roles. A particular focus on the timing and extent of this evolution must be made, so as to capitalize on areas of potential while safeguarding from harmful steps that could challenge progress toward quality clinical care.

## Task-Shifting to Non-physician Clinicians

Across many developing countries, NPCs now fill physician-deficit gaps in healthcare delivery, creating the Eyal et al termed “NPC-cornerstone system,” which the authors claim can function with less physicians.^1^ As Eyal et al write, many developing countries in sub-Saharan Africa have artfully used task-shifting and decentralization to capitalize on existing human resource capacity within the health sector.^3^ In Rwanda, task-shifting occurs between all health professionals in order to counter shortages across every skilled position. At the community level, nurses manage infectious disease effectively through this decentralized transfer of select medical procedures.^4^ This has enabled care to be delivered more efficiently, vastly, and at a lower-cost than the traditional physician-driven system, resulting in the improvement of the healthcare system as a whole.^5^

## Clinical Specialization – A Needed Evolutionary Step

Based on experience, we agree with Eyal et al that, at each task-shifted-care level, certain areas cannot be transferred and physicians must retain critical, specialized roles. Consider Rwanda’s current physician ratio of 678 medical doctors: 191 are specialists, with only 2 oncologists (Ministry of Health of the Government of Rwanda, Unpublished data, 2016). If either of these 2 oncologists fully transitions into a leadership position or a research role, only 1 Rwandan oncologist will be left to treat the country’s entire cancer population.

## Non-clinical Education Makes More Well-Rounded Physicians

Eyal et al advocate for a parallel evolution in education to prepare doctors for new, non-clinical opportunities.^1^ Time gained as a result of task-shifting can now, the authors argue, be used to increase physician training in specialized medical fields as well as non-clinical domains such as: health service delivery, workforce capacity building, research, information systems, health financing, ethics, leadership and governance. Given the physician density across sub-Saharan Africa though, such education would be effective if, and only if, it is directly tied to clinical care. This will empower physicians to understand how to improve service delivery on an individual, team, and facility level and to provide health sector governance leaders with relevant feedback.^6^ No longer in clinical silos as they now increasingly lead multidisciplinary teams, physicians’ mastering of management skills will ultimately benefit patients as well.^7^

Yet in order to teach non-clinical skills that may strengthen physicians’ clinical work, a decision about timing and education placement must be made. Here, we disagree with the proposal by Eyal et al for a “reduction in coursework that is [in the authors’ opinion] less relevant to health service delivery in resource-poor settings” in order to introduce non-clinical skills.^1^ This reduction threatens to create substandard medical education, which would not enable the future system to function at its highest clinical capacity for those it should care for most: the patients. Populations in developing countries face the same medical conditions as developed countries; thus, the same, high-quality specialized care is needed in both. We, therefore, advocate that education in non-clinical knowledge must only be put in place if it improves clinical medical skills and education, in lieu of reducing or replacing it.

Human Resources for Health (HRH) exemplifies this notion of a strategic and comprehensive program that strengthens both clinical and non-clinical domains. Building capacity through service delivery and in-service mentorship, while concurrently sustainably evolving select areas in advanced medical education, HRH in Rwanda demonstrates an innovative, well-designed twinning program in a developing country.^8^

## Non-physician Clinician-Education: A Need for Standardized, Formal Training

Eyal et al propose that in a NPC-based health system, physicians are well-suited to train NPCs in diagnostic and clinical skills. However, it seems more appropriate and effective to create formal, pre-service NPC Technical and Vocational Education and Training (TVET) programs through the education sector. Such short, cost-effective instruction would ensure standardization and consistency in the quality of NPCs.^9^ In Rwanda, this structured education has been done with the Home Base Care Practitioners (HBCP) program, in which the Ministry of Education gives a 6-month TVET education, ensuring standardization, quality, and consistency.

## Physicians as Non-physician Clinician Mentors: An Effective Evolutionary Step

Eyal et al put forth the notion that supervision and mentoring may diminish physicians’ resistance to NPCs and ensure consistency in the quality of services delivered. In the HBCP-TVET program, specialized physicians and nurses conduct mentorship over the new NPCs. Supervisory standards ensure that task-shifting upholds high clinical standards, diminishing mentorship inconsistencies that could negatively impact patients. Through structured education rather than individualized, physician-led instruction, we can ensure that all NPCs have the same knowledge. Guided supervision and mentorship roles create an opportunity for physicians to take on purposefully selected non-clinical work in a manner that directly impacts clinical care, while also encouraging physician-NPC trust through improved inter-professional relationships.^7^

## How to Tackle Internal Migration, the Clinical Brain Drain?

Eyal et al highlight two physician “brain drains:” one in-country migration from clinical roles to leadership or private opportunities, and another drain from developing countries to positions aboard with higher pay and increased opportunities.^10^ We agree with Eyal et al that the first, in-country brain drain can effectively be countered by the promotion of NPCs to decrease the burnout of the few doctors available.^11^

For the second brain drain – from local to international positions – we propose three possible solutions:

1. Continue to increase the number of people receiving quality medical education, so as to train a critical mass of physicians. This will ensure that, in the future, those advanced clinical services that NPCs cannot perform are sufficiently delivered to the population safely and comprehensively.
2. A system of individual incentives must be put in place to retain in-country physicians in the clinical sphere. Government-led programs, such as performance-based financing and tax exemption incentives, have been integrated in Rwanda for this specific purpose.^12^
3. To protect developing countries’ human resource development, a system of institutional or country-compensated penalties, similar to that used by athletic organizations to police recruitment of under-contract personnel, must be put in place.^13^ This solution will target both individuals and recruiting nations or companies who use their wealth to incentivize doctors to abandon their clinical practice in developing countries. Through this penalty system, international brain drain will be discouraged, the lost human resource value developing countries experience will be countered, and the financial means for countries to educate new physicians will be availed.^14^

## Needed Boundaries: Recruiting From Outside the Medical Field

In the midst of these discussions around physician evolution, we must remain keenly aware of current health systems. Across sub-Saharan Africa, physicians with clinical expertise but no formal, non-clinical training are often appointed as Hospital Director or Division Head within Ministries of Health in reaction to management shortages.^15^ Rather than taking physicians from already-strained clinical spheres, it would be more advantageous to educate non-clinical professionals through hospital administration or health management advanced degrees. By occupying these positions in this way, the systems would be strengthened without leaving clinical gaps where physicians are still greatly needed.^10^ Through a Masters degree in Hospital Administration (MHA) at the University of Rwanda, non-professionals from outside of the traditional medical field can now step into the health sector, bolstering its management and administration and allowing physicians to continue in the medical roles they were trained to fill.^8^

## A Cautious, Calculated Evolution: A Way Forward

Studies have shown that NPCs can perform certain designated clinical functions at an equally safe and medically sound quality to physicians. We encourage health education across the developing world to adapt and promote NPC education with quality, strict standards such as those present in the TVET for HBCP.^5,9,16^ Yet in the context of still-remaining significant physician deficits, we question if NPCs can support the clinical sphere to the degree that would safely allow physicians to move beyond the service domain. There remain far too few physicians available for a percentage to provide and develop clinical practice while concurrently evolving into specialization training and non-clinical areas.^17^ The bottom line is clear: future physicians should not exit the clinical space when life-saving clinical skills are still needed most.^2^

While we disagree with the proposition by Eyal et al to evolve clinicians across non-clinical fields, we agree with their proposal to evolve physicians in purposefully selected specialized areas. An increase in the number of highly trained specialty physicians is crucial in order to answer to the ever-changing epidemiological shifts occurring in already underserved populations.^5^

Around the world, the provision of quality care will only occur if there is collaboration between all qualified healthcare providers and if evolution of any roles takes place in a timely, contextually sensitive manner. Accelerating the attainment of a system in which all people have the right to access and receive the needed quantity of quality health services, this collaboration and sensitivity will encourage systems to be in line with national and international education and medical standards. The development of this integrated health system, in which both physicians and NCPs each have their place in achieving the evolving goal of quality care for all, is, for now, the utmost priority.

## Ethical issues

Not applicable.

## Competing interests

The authors declare that they have no competing interests.

## Authors’ contributions

AB and AE contributed equally to developing the manuscript. AB, GS, and AE contributed equally to revising and reviewing the manuscript.

## Authors’ affiliations

^1^Minister of Health, Kigali, Rwanda. ^2^Harvard Medical School, Boston, MA, USA. ^3^Geisel School of Medicine, Dartmouth University, Hanover, NH, USA. ^4^University of Global Health Equity, Kigali, Rwanda. ^5^University of Vermont, Burlington, VT, USA. ^6^The Dartmouth Institute of Health Policy and Clinical Practice, Hanover, NH, USA.
